# Supplementary material for: Skeletal muscle atrophy and myosteatosis are not related to long-term aneurysmal subarachnoid hemorrhage outcome
Source: PLoS One. 2022 Mar 4;17(3):e0264616. doi: 10.1371/journal.pone.0264616 (PMC8896675; doi:10.1371/journal.pone.0264616)
Supplement: Attachment — Submitted filename: Response to Reviewers_final.docx [file pone.0264616.s001.docx]

**Response to review 1**

1. *Should be added more detail about the in-house developed software (SarcoMeas 0.34; UMCG, Groningen, The Netherlands) as described in the Vedder IR, Levolger S, Dierckx RAJO, et al. Effect of muscle depletion on survival in peripheral arterial occlusive disease: Quality over quantity. J Vasc Surg. 2020;72(6):2006- 2016.e1. doi:10.1016/j.jvs.2020.03.050.*

In the methods section of the revised manuscript, we have added information (underlined sentences) regarding the software package that was used to segment and measure the muscles:

**Page 4, line 127-136, methods section:** *“*In-house developed software (SarcoMeas Neck 0.34; UMCG, Groningen, The Netherlands) was used to perform the skeletal muscle measurements. With this software, the neck muscles at the level of the third cervical vertebra were manually delineated by one investigator (AK) based on radiodensity as expressed in Hounsfield Units (HU). The target muscles are illustrated in Figure 1. Within these contours, voxels with a radiodensity from -29 to 150 HU were identified as skeletal muscle. The Skeletal Muscle Area (SMA) was determined by calculating the volume of all muscle voxels. The SMA was then corrected for patient length, by dividing the muscle area by the squared patient length, resulting in the Skeletal Muscle Index (SMI), expressed in cm^2^ /m^2^. Skeletal Muscle Density (SMD) was defined as the mean density of all muscle voxels within the drawn contours.*”*

2. *The authors mention that “Both interobserver and intraobserver levels of agreement for SMA and SMD were found to be excellent in a prior study”, however there might be study-specific variations as the Vedder et al assessed patients with peripheral arterial occlusive disease while the current study assess cross-sectional muscle measurement in the cervical region. Taking into account the manual segmentation.*

In a previous study the interobserver and intraobserver levels of agreement of the software package was indeed investigated. The reviewer is correct that this was however at the level of the third lumbar body. We have therefore determined the interobserver and intraobserver reliability for measuring skeletal muscle atrophy and myosteatosis at the level of the third cervical vertebra. Text regarding the methodology and results has been added to the revised manuscript.

**Page 4, line 137-139, methods section:** “To assess interobserver variability, all measurements were independently repeated by a second investigator (AV). Moreover, 50 random cases were remeasured by the first investigator (AK) after two years, for assessment of intraobserver agreement.”

**Page 7, line 203-204, results section:** “Both interobserver and intraobserver levels of agreement for SMA and mean HU value were found to be excellent (Table 2).”

Table 2. Interobserver and intraobserver levels of agreement for SMI and mean HU value

| Agreement* | Skeletal Muscle Area | Mean HU |
| --- | --- | --- |
| Interobserver | 0.988 (95%CI 0.984, 0.991) | 0.995 (95%CI 0.990, 0.997) |
| Intraobserver | 0.975 (95%CI 0.884, 0.991) | 0.908 (95%CI 0.832, 0.949) |

* Intraclass correlation coefficient, absolute agreement, two-way random, average measures

3. *It's unclear how muscle atrophy was defined by a sex-specific cutoff value and how myosteatosis was defined by a BMI-specific cutoff value. Should be explain in the method section. Should be added the background why these specific thresholds for skeletal muscle and for intra- and inter-muscle adipose tissue have been selected.*

The rational for establishing sex- and BMI-specific thresholds is that from previous literature it is known that the average muscle mass in male and female are different and that muscle density varies according to BMI. To enhance clarity, we have rephrased the methods section of the revised manuscript:

**Page 5-6, line 156-173, statistical analysis section:** “The presence of myosteatosis was determined by means of applying a threshold. From previous studies it is known that the threshold of myosteatosis varies significantly based on the BMI of the subject [1,2]. Therefore, we established BMI-specific cutoff points of mean HU value for myosteatosis by optimum stratification. Optimal stratification is a strategy based on log-rank statistics to determine at which cutoff values the most significant difference would occur with regard to a binary outcome or a time-to-event outcome, such as death in this study [3]. A two-step procedure was performed to determine the thresholds: First, all patients were split into two subgroups based on the value of BMI 25kg/m^2^ according to the WHO definition of overweight [4]; Secondly, within each subgroup, the mean HU value with the most significant P value for overall mortality by log rank statistics was selected as the cutoff point of the BMI-specific group.

Muscle mass in males is furthermore known to differ significantly from that in females [5]. To determine the presence of muscle atrophy, we corrected for this by applying a sex-specific threshold for muscle atrophy in accordance to previous studies [2]. An attempt was made to determine the SMI threshold following the two-step procedure as stated above, however a threshold was not found within this cohort. Previously reported sex-specific cutoff points for sarcopenia were therefore used (7.8 cm^2^/m^2^ for female, and 12.1 cm^2^/m^2^ for male) [6].”

4. *As aging is associated with myosteatosis, it might be the effect of aging that the myosteatotic group in this study are more likely to be above the age of 70.*

The reviewer raises a valid point and age was indeed taken into account. In our study we used a cox proportional hazards model to assess the correlation between muscle alternations and mortality. Given that there were more patients 70 years or older in the myosteatosis group than non-myosteatosis group (table 1; 31% vs 9.4%, p<0.001) and that age was associated with death in the univariable analyses, age was added as covariable in the multivariable analyses. The co-variables used in the cox-proportional hazards model are reported in the legend of Table 3.

5. *As a limitation, it should be highlight that standardization of CT-derived diagnostic thresholds for muscle mass and myosteatosis is lucking.*

In the limitation section of the revised manuscript, we have accordingly added text (underlines sentences) regarding the lack of standardization.

**Page 14, line 336-340, discussion section: “**Another limitation of this study is that for CT based measurements of muscle atrophy and myosteatosis, there are no standardized thresholds. In this study we have therefore performed optimal stratification to determine the threshold for myosteatosis and adopted a previously reported threshold for muscle atrophy, however further external validation is required to investigate whether these measures are generalizable.”

6. *Table 1: what is the rational behind the variables age > 70y and BMI < 25. Should be stated in the material and methods section.*

Literature has shown that an age of 70 or older is an independent risk factor for poor neurological outcome 2 month after aneurysmal SAH [7]. We have therefore dichotomized age by 70 years. Furthermore, according to WHO guidelines, people with BMI higher than 25 kg/m^2^ are considered as overweight [4], thus we dichotomized BMI by 25 kg/m². For clarity we have added text the methods section of the revised manuscript

**Page 5, line 179-180, statistical analysis section**: “As an age of 70 or older is an independent risk factor for poor neurological outcome 2 month after aneurysmal SAH, age was dichotomized at 70 years.[21]”

**Page 5, line 162-164, statistical analysis section**: “A two-step procedure was performed to determine the thresholds: First, all patients were split into two subgroups based on the value of BMI 25kg/m^2^ according to the WHO definition of overweight [4].”

 General comments

7. *References need to be homogenized, for example, introduction section line 51.*

Thank you for pointing out this error. We have corrected the references in the revised manuscript.

8. *Typos line 77 (material and methods), line 118 (figure 1 legend)*

The typographical errors within the manuscript have been changed accordingly. We have further thoroughly checked the remainder of the manuscript for errors.

9. *Figure 1, color names in the figure legend not correspond to color in the figure*

Thank you for pointing out this error, we have corrected the color name as cyan in the revised manuscript at line 117.

10. *Table 1: is too busy to read*

To improve clarity, we have revised the table and transferred it to a landscape view.

**Response to review 2**

*In this retrospective study, authors evaluated the correlation between sarcopenia or myosteatosis and poor outcome after subarachnoid hemorrhage due to aneurysmatic rupture. They did not find statistically significant results with mortality and poor neurological function.*

*Overall, the manuscript is sound and clearly written; however, authors could improve the section of Methods according to the following suggestions.*

1. *Details about the CT protocol parameters*

In the revised manuscript, we have added extra information regarding the CT protocol parameters:

**Page 3, line 104-106:** “CT-imaging was performed on a Siemens SOMATOM Definition Edge or SOMATOM Sensation (Siemens Medical, Erlangen, Germany), using a 512x512 matrix, soft-tissue reconstruction kernel and a slice thickness of 3-5 mm.”

2. *Could the thickness variability (1 to 5 mm) have influenced the cross-sectional muscle measurements?*

Based on the comment of the reviewer, we individually assessed the slice thickness throughout all patients. Of the 293 subjects, 289 (98.63%) had a slice thickness of 3 mm. Given that the slices varied only in 4 patients, we do not expect that this may have influenced the muscle measurements.

3. *Number of investigators in charge of muscle mass measurement*

Extra information regarding the segmentation of the muscles has been added to the methods section of the revised manuscript at **Page 4, line 127-136, methods section**. We also would like to refer to the first question of the first reviewer.

**Reference**

1	Martin L, Birdsell L, MacDonald N, *et al.* Cancer cachexia in the age of obesity: Skeletal muscle depletion is a powerful prognostic factor, independent of body mass index. *J Clin Oncol* 2013;31:1539–47. doi:10.1200/JCO.2012.45.2722

2	Vedder IR, Levolger S, Dierckx RAJO, *et al.* Effect of muscle depletion on survival in peripheral arterial occlusive disease: Quality over quantity. *J Vasc Surg* 2020;72:2006-2016.e1. doi:10.1016/j.jvs.2020.03.050

3	Williams BA, Mandrekar JN, Mandrekar SJ, *et al.* Finding Optimal Cutpoints for Continuous Covariates with Binary and Time-to-Event Outcomes Department of Health Sciences Research Mayo Clinic Rochester , Minnesota Copyright 2006 Mayo Foundation. 2006.

4	World Health Organisation. Obesity and overweight. Fact sheet N°311. Fact sheet N. 2013.

5	So JL, Janssen I, Heymsfield SB, *et al.* Relation between whole-body and regional measures of human skeletal muscle. *Am J Clin Nutr* 2004;80. doi:10.1093/ajcn/80.5.1215

6	Ufuk F, Herek D, Yüksel D. Diagnosis of sarcopenia in head and neck computed tomography: Cervical muscle mass as a strong indicator of Sarcopenia. *Clin Exp Otorhinolaryngol* 2019;12:317–24. doi:10.21053/ceo.2018.01613

7	Van Donkelaar CE, Bakker NA, Birks J, *et al.* Prediction of Outcome After Aneurysmal Subarachnoid Hemorrhage: Development and Validation of the SAFIRE Grading Scale. *Stroke* 2019;50. doi:10.1161/STROKEAHA.118.023902
